# Development of nanobody-based DAS-ELISA and Au nanoparticle-based immunochromatographic test strip for highly sensitive detection of Chikungunya virus

**DOI:** 10.3389/fimmu.2025.1707358

**Published:** 2025-11-18

**Authors:** Mengmeng Guo, Liyuan Song, Jingliang Liu, Yating Hu, Yihui Chen, Guangcheng Fu, Hongyu Yuan, Jianmin Li

**Affiliations:** 1 Laboratory of Advanced Biotechnology, ZJU-Hangzhou Global Scientific and Technological Innovation Center, Zhejiang University, Hangzhou, China; 2 Laboratory of Advanced Biotechnology, Beijing Institute of Biotechnology, Beijing, China

**Keywords:** Chikungunya virus, immune nanobody library, nanobody, DAS-ELISA, AuNP-ICTS

## Abstract

**Introduction:**

Chikungunya virus (CHIKV) is a mosquito-borne alphavirus that causes severe disease in humans and animals and continues to pose a significant global public health threat. Current diagnostic methods primarily rely on detecting CHIKV-specific IgM and IgG antibodies; however, these methods are limited to the humoral immune phase following viral infection.To address the diagnostic gap during the window period before antibody response, we developed two antigen detection assays capable of directly detecting the CHIKV envelope protein (CHIKV-E), enabling early and rapid viral detection.

**Methods:**

In this study, 20 nanobodies (Nbs) specifically binding to CHIKV-E protein were screened and identified from a constructed immune nanobody library using phage display technology. These Nbs were fused to human IgG1-Fc and expressed as recombinant Nb-Fc antibodies in Expi293F cells. Subsequently, the preferred Nb-Fc pairs (N055-Fc/N055-mIgG1-Fc and 10G4-Fc) were used to establish double-antibody sandwich ELISA (DAS-ELISA) and Au nanoparticle-based immunochromatographic test strip (AuNP-ICTS) assays.

**Results:**

Both the DAS-ELISA and AuNP-ICTS demonstrated high sensitivity for CHIKV-E. The two assays also exhibited exclusive specificity for CHIKV-E, showing no cross-reactivity with envelope proteins from four related alphaviruses or two co-circulating flaviviruses. Epitope mapping revealed that nanobodies N055 and 10G4 recognized distinct linear epitopes: N055 targets E1 Domain II, whereas 10G4 recognizes the E1 fusion loop with additional interactions in E1 Domain II and E2 Domain B. Quantitative sensitivity analysis indicated that both DAS-ELISA and AuNP-ICTS effectively detect CHIKV-E in serum, with limits of detection (LoD) of 49 pg/mL and 1.56 ng/mL, respectively-demonstrating superior sensitivity compared with existing antigen detection methods. Notably, the AuNP-ICTS method completes detection within 10 min, significantly improving efficiency.

**Discussion:**

Collectively, the developed DAS-ELISA and AuNP-ICTS assays overcome the limitations of conventional IgM/IgG serological methods, providing superior sensitivity and rapid detection capabilities that make them promising diagnostic tools for early CHIKV detection.

## 1 Introduction

Chikungunya virus (CHIKV), a member of the *Alphavirus* genus (*Togaviridae* family), is the causative agent of Chikungunya fever and a re-emerging pathogen that poses a significant threat to global public health (1, 2). Alphaviruses are enveloped viruses transmitted primarily by mosquitoes that cause severe disease in humans and animals, with clinical manifestations ranging from fever and rash to severe inflammatory lesions, including encephalitis and arthritis. Alphaviruses can be broadly categorized into two groups based on disease presentation (1): neurotropic alphaviruses, such as Eastern equine encephalitis virus (EEEV), Western equine encephalitis virus (WEEV), and Venezuelan equine encephalitis virus (VEEV) (3), which primarily invade the nervous system and cause encephalitis (4, 5) and arthritogenic alphaviruses, exemplified by Mayaro virus (MAYV) and CHIKV, which predominantly cause acute and chronic arthritis (6). CHIKV is primarily transmitted by Aedes aegypti and Aedes albopictus mosquitoes (7, 8), with frequent geographic and temporal overlap with MAYV, dengue virus (DENV), and Zika virus (ZIKV) transmission. This co-circulation creates diagnostic challenges due to similar clinical presentations; moreover, serological tests (IgM/IgG) for MAYV, DENV, ZIKV, and CHIKV suffer from cross-reactivity, further complicating diagnosis (9, 10). Patients infected by CHIKV typically present with high fever (≥39 °C), often accompanied by rash and severe arthralgia and myalgia that can persist for years. In addition, viremia occurs simultaneously with fever and peaks in the acute phase (lasting approximately 1 week), with viral loads up to 10^9^ genome copies/mL of blood (11). Severe cases may progress to encephalitis, myocarditis, hepatitis, or multiorgan failure (12, 13).

The CHIKV virion is spherical, approximately 70 nm in diameter. The inner layer of the virion consists of a positive-sense single-stranded RNA genome wrapped by an icosahedrally symmetric nucleocapsid structure composed of 240 capsid proteins. The RNA genome of CHIKV is approximately 11.8 kb in length and contains two discontinuous open reading frames (ORFs). The 5’-terminal ORF encodes four nonstructural proteins (nsP1–4) associated with the viral replication complex, whereas the 3′-terminal ORF encodes five structural proteins (C, E3, E2, 6K, and E1) (14). The nucleocapsid is surrounded by a host-derived lipid bilayer. The outermost layer of the virion is an envelope structure composed of three structural proteins (E1, E2, E3) that assemble at the plasma membrane to form 240 E1–E2 heterodimers, which in turn assemble into 80 heterotrimeric spike glycoproteins arranged in icosahedral symmetry with T = 4 on the virion surface (15, 16). Glycoprotein E2, derived from furin cleavage of the p62 precursor into E3 and E2, mediates receptor binding and viral entry via endocytosis; E3 is mainly involved in the assembly of spikes; and E1 contains fusion peptides that, at low pH, change the conformation of the E1–E2 heterodimer, exposing the epitopes to facilitate membrane fusion and allow nucleocapsid release into the host cytoplasm (17, 18). In addition, E1 and E2, as the primary surface-exposed proteins, exhibit strong immunogenicity and serve as dominant targets for the host immune response. The precursor p62–E1 protein (E3–E2–E1) maintains the native-like conformation of CHIKV envelope proteins. The E3 protein facilitates proper folding of E2 and E1 and preserves critical conformational epitopes at the E2–E1 interface. These characteristics make p62–E1 a superior immunogen compared with isolated recombinant E2 or E1 proteins (16).

Nanobody (Nb), also known as VHH (variable domain of the heavy chain of heavy-chain antibody), constitutes a groundbreaking class of single-domain antibodies derived from camelid heavy-chain antibodies (HCAbs) discovered in 1993 (19). Nbs represent a fully functional antigen-binding fragments with a molecular mass of approximately 15 kDa. Moreover, Nbs exhibit several distinctive advantages, including low immunogenicity, excellent biocompatibility, and high binding affinity (typically in the nanomolar range). Structural analyses reveal that Nbs possess extended complementarity-determining region 3 (CDR3) loops (16–24 residues versus 8–15 in conventional antibodies), which enable them to enhance interaction with antigens by penetrating deeper into the antigenic epitope (20). In addition, the simple single-domain structure of Nbs makes them easy to genetically modify, and their high sequence homology with human VH (70%–80%) allows them to be directly humanized with appropriate modifications and optimization. These distinctive properties provide Nbs and their recombinant derivatives with unique advantages in diverse applications, including (1) targeted cancer therapies, (2) viral neutralization platforms, and (3) advanced diagnostic systems (21, 22).

Since CHIKV was first described during an outbreak on the Makonde Plateau in Tanzania, East Africa (1952–1953) (23), it has spread to sub-Saharan Africa, Europe, the Americas, and Asia, causing multiple large-scale epidemics and resulting in significant public health and economic burdens (24, 25). In addition, the expanding geographic range of Aedes aegypti and Aedes albopictus mosquitoes, driven by climate change and globalization, further exacerbates its global transmission risk (26, 27). Given the ongoing threat of CHIKV, early surveillance and strict quarantine measures are crucial for the rapid control of the epidemic (24). Currently, CHIKV detection primarily relies on the identification of CHIKV-specific IgM and IgG (28–30). Serum IgM can be detected from 5 days to several months after symptom onset during acute infection, whereas IgG appears 1–2 weeks after onset (usually post-viremia) and persists for months to years, indicating convalescent or past infection (13). Antigen-based detection could offer a solution by targeting viral proteins directly and overcoming the window-period limitation of specific IgM/IgG detection, enabling early and rapid diagnosis.

Several antigen detection assays for CHIKV have been reported to date. These methods include the indirect enzyme-linked immunosorbent assay (indirect-ELISA) (31), double antibody sandwich enzyme-linked immunosorbent assay (DAS-ELISA) (32–34), fluorescent immunosorbent assay (FIA) (33), time-resolved fluor immunoassay (TRFIA) (35) and immunochromatographic test strips (ICTS) (32, 36). Among these, ICTS, also known as lateral flow assays, are extensively applied in diverse fields, including medical diagnostics, environmental monitoring and point-of-care testing (POCT), owing to their rapid visual readout (typically within 10–20 min), low cost, and minimal dependence on specialized equipment or reagents (37, 38). Currently, Au nanoparticle-based ICTS are the most widely used format in rapid diagnostics, primarily due to the unique advantages of AuNPs as both signal reporters and antibody carriers (39). AuNPs have emerged as a prominent tool in bioanalytical applications owing to their exceptional optical properties, robust stability, and straightforward conjugation chemistry (39, 40). Their outstanding biocompatibility allows them to preserve the native activity of biomolecules, such as antibodies, antigens, and nucleic acids, through electrostatic adsorption. Additionally, the ease of synthesis and cost-effectiveness of AuNPs make them an ideal choice for lateral flow immunoassays, contributing to their extensive implementation in rapid diagnosis technologies (41).

In this work, we describe the use of phage display technology to obtain nanobodies against CHIKV to develop highly sensitive diagnostic tools such as DAS-ELISA and Au nanoparticle-based immunochromatographic test strips (AuNP-ICTS). The sensitivity of DAS-ELISA and AuNP-ICTS for detecting CHIKV envelope protein in human serum was 49 pg/mL and 1.56 ng/mL, respectively, which is significantly higher than that of other current antigen detection studies. In addition, the developed AuNP-ICTS could detect CHIKV envelope protein in human serum within 10 min. Both assays exhibited exclusive specificity for CHIKV E protein without cross-reactivity to envelope proteins of related alphaviruses or co-circulating flaviviruses. Our results indicate that both developed assays exhibit high specificity and sensitivity in detecting CHIKV antigens.

## 2 Materials and methods

### 2.1 The CHIKV envelope protein expression and purification

The CHIKV-E (p62–E1, including E3–E2–E1) protein gene sequence (GenBank: MH670649.1) was optimized based on the codon preferences of insect cells, and six histidine (His) residues were added to the C-terminal of the gene sequence. The optimized gene sequence with the His tag was synthesized by artificial methods. The synthetic gene fragments were then seamlessly cloned into the pFastBac1 vector to obtain recombinant plasmids, which were transformed into DH10Bac competent cells. Blue–white screening and PCR analysis were subsequently performed to obtain the recombinant baculovirus particles (Bacmid). The recombinant baculovirus particles were extracted using the precipitation method with isoamyl alcohol. Subsequently, the Bacmid was transfected into ExpiSf21 insect cells (Thermo Fisher Scientific, Waltham, MA, USA) using the ExpiFectamine™Sf Transfection Reagent (Gibco, Carlsbad, CA, USA). The cell culture supernatant was collected after 4 days of incubation. The centrifuged supernatant (CHIKV-E P0 virus) was then transfected into ExpiSf9 cells for viral replication. Five days post-transfection, the cell supernatant was collected and purified using a HisTrap™ Excel affinity chromatography column (Cytiva, Uppsala, Sweden). The concentrated CHIKV-E protein was finally identified via SDS–PAGE. Protein concentration was estimated by measuring UV absorbance at 280 nm using a NanoDrop™ One/One^C^ spectrophotometer (Thermo Fisher Scientific, Waltham, MA, USA).

### 2.2 The immune nanobody library construction

To obtain anti-CHIKV nanobodies (Nbs), an immune Nb library was constructed. The purified CHIKV-E protein was used to immunize an alpaca through multipoint subcutaneous injection at 3-week intervals. Briefly, the alpaca was immunized with 1 mg of purified CHIKV-E protein emulsified in complete Freund’s adjuvant (Sigma-Aldrich, St. Louis, MO, USA). After the fifth immunization, the anti-CHIKV-E IgG titer in serum was tested using the ELISA method. CHIKV-E protein (2 µg/mL in PBS) was coated on a 96-well plate (100 µL/well) overnight at 4°C. After washing with PBST (pH 7.4), the plate was blocked with 3% bovine serum albumin (BSA) (Sigma-Aldrich, St. Louis, MO, USA) in PBS (200 µL/well) for 1 h. Serially diluted alpaca serum (in PBST/3% BSA) was added to the plate (100 µL/well) and incubated for 1 h at 37°C. Following PBST washes, bound antibodies were detected using HRP-conjugated anti-alpaca IgG antibody. The immunized alpaca developed a final titer of 1×10^6^, demonstrating successful humoral immune response induction by the CHIKV-E antigen. Peripheral blood mononuclear cells (PBMCs) were then purified from extracted blood for mRNA extraction and cDNA synthesis. Total RNA was extracted from the isolated PBMCs using the MiniRNA extraction kit (QIAGEN, Hilden, Germany). The total RNA was used as a template to synthesize a cDNA library with the Super Script-III First-Strand Synthesis System kit (Invitrogen, USA), according to the manufacturer’s instructions. The cDNA library was used as a gene template, and primers CALL001 (5’-GTCCTGGCTGCTCTTCTACAAGG-3’) and CALL002 (5’-GGTACGTGCTGTTGAACTGTTCC-3’) were used for the first round of PCR. The first-round products were then used as templates for a second PCR amplification employing primers VHH-FOR(5’-GATGTGCAGCTGCAGGAGTCTGGRGGAGG-3’) and VHH-REV (5’-TAGTGCGGCCGCTGAGGAGACGGTGACCTGGGT-3’). Subsequently, the obtained amplicons were ligated into the linearized pComb3X vector using T4 ligase.

**Figure 1:**
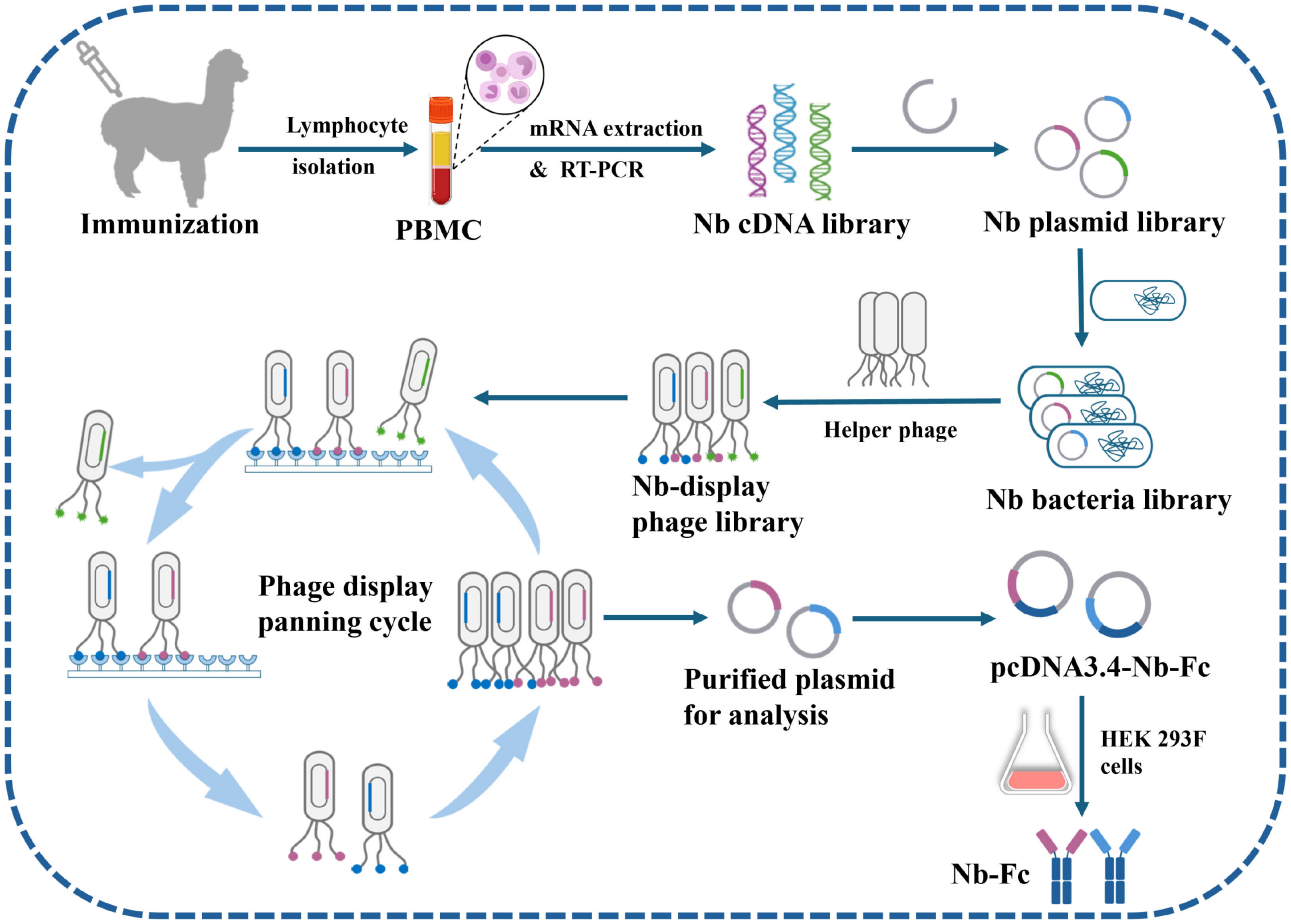
Schematic representation of screening CHIKV-specific nanobodies (Nbs) from an immune nanobody library. The alpaca is inoculated with CHIKV-E protein, blood is collected, lymphocytes are extracted, mRNA is isolated, and an Nb cDNA library is constructed via two rounds of PCR. The library is inserted into phage vectors and transformed into *E. coli* cells to produce phages containing the Nb nucleotide sequence and displaying the Nb on their surface. High-affinity Nb phages are then isolated through multiple rounds of panning. These Nb candidates are sequenced, modified, and inserted into the pcDNA3.4 vector, and the HEK 293F cell expression system is used to produce Nbs.

The recombinant plasmids were transformed into electrocompetent *E. coli* XL1-Blue cells, resulting in a nanobody bacterial library with 5.52 × 10^8^ independent transformants. The library titer was determined to be 4.9 × 10^8^ through sequencing analysis of selected monoclonal colonies, fulfilling the criteria for high-quality library construction. The XL1-Blue library stock was subsequently infected with VCSM13 helper phages to generate a phage display library presenting nanobodies (**Figure 1**).

### 2.3 Phage display for screening of anti-CHIKV Nbs

The Nb-display phage library was generated by infecting the bacterial library with VCSM13 helper phages. After amplification and 0.22 μm filtration, the phage library achieved a titer of 2.34 × 10¹²/mL, as quantified by serial dilution analysis. Subsequently, the phage library was subjected to indirect ELISA screening to isolate CHIKV-specific Nbs.

Phages displaying Nbs on their coat proteins were introduced into high-affinity 96-well ELISA microplates coated with CHIKV-E protein and incubated for 2 h at 37 °C. After rinsing the plates three times with 0.01 M phosphate-buffered saline containing 0.05% Tween-20 (PBST, pH 7.4), glycine–HCl (100 µL/well) was added and incubated at 37°C for 10 min to elute positive phages. The eluted phages were rapidly neutralized with an equal volume of 1 M Tris–HCl (pH 7.4). The obtained positive phages were then transfected into new *E. coli* XL1-Blue cells, shaken at 37°C for 30 min, and rescued by incubation overnight at 30°C with shaking to obtain the amplified first Nb library. The above procedure was repeated to construct the second Nb library, increasing the Tween-20 concentration to 0.2% and the number of washes to six to enhance selection stringency. Following two rounds of phage display panning, significant enrichment of specific phages was observed, with the output/input phage titer ratio increasing from 10^-5^ (Round 1) to 10^-2^ (Round 2). A total of 96 monoclonal phages were randomly selected from bacterial plates after the second round of screening. After IPTG induction, high-affinity Nb phages were prepared, and their reactivity with CHIKV-E protein was initially validated using phage ELISA. When the OD_450_ value of a clone was more than three times that of the PBS control, it was judged as positive. The selected positive clones were then sequenced to determine their Nb nucleotide sequences (**Figure 1**).

### 2.4 Generation of anti-CHIKV Nb-Fcs

To obtain high expression of anti-CHIKV Nbs and enhance their stability, the core coding sequences of the Nbs were appended with human IgG1-Fc sequences at the C-terminal end and ligated into the pcDNA3.4 vector via restriction enzyme recognition sites Eco RI and Eco RV. The recombinant plasmids were transfected into Expi293F cells (Thermo Fisher Scientific, Waltham, MA, USA) using the Expifectamine™ 293 Transfection Kit (Gibco, Carlsbad, CA, USA) and incubated at 37°C for 5 days.

The supernatant was collected, centrifuged at 8,000 rpm for 15 min, and filtered through a 0.22 µm syringe filter. The recombinant nanobodies (Nb-Fcs) were purified using a 5 mL HiTrap™ Protein A HP column (Cytiva, Uppsala, Sweden) (binding buffer: PBS, pH 7.5; elution buffer: 0.1 M glycine, pH 2.7). The purified Nb-Fcs were concentrated using an ultrafiltration centrifuge tube with a cut-off molecular weight of 15 kDa, and the buffer was replaced with PBS (pH 7.4). The expression and purity of the Nb-Fcs were confirmed by reduced SDS–PAGE.

### 2.5 Characterization of the anti-CHIKV Nb-Fcs

The binding activity EC_50_ (half-maximal effective concentration) between the Nb-Fcs and CHIKV-E protein was estimated by indirect ELISA using purified CHIKV-E protein as the coating antigen. CHIKV-E protein was diluted to 2 µg/mL in sodium bicarbonate buffer (pH 9.6) and coated onto 96-well ELISA microplates (100 μL/well), then incubated overnight at 4°C. After three rinses with PBST (pH 7.4), all wells were blocked with 0.01 M PBS containing 3% BSA (Sigma-Aldrich, St. Louis, MO, USA) (200 μL/well) for 60 min.

Each anti-CHIKV Nb-Fc solution was then serially diluted threefold from 3 µg/mL across the horizontal rows of the plate and incubated for 1 h at 37 °C. After three washes, HRP-conjugated mouse anti-human IgG antibody was added to all wells and incubated at 37 °C for 60 min. After washing, 50 µL of TMB (3, 3’, 5, 5’-tetramethylbenzidine) substrate was added to each well for 10 min, followed by 2 M H_2_SO_4_ to stop the reaction. The optical density at 450–630 nm (OD_450-630_) of each well was immediately measured with a microplate reader. Finally, a standard curve was plotted using the four-parameter logistic model to analyze the EC_50_ value of the obtained Nb-Fcs. Specificity and binding epitopes of the Nb-Fcs to CHIKV were validated by western blot.

### 2.6 Establishment of DAS-ELISA for CHIKV detection

Purified Nb-Fcs against CHIKV were conjugated with horseradish peroxidase (HRP) using an HRP Labeling Kit (Biodragon) according to the manufacturer’s instructions. The titers of the HRP-conjugated Nb-Fcs (HRP–Nb-Fcs) were determined by indirect ELISA using CHIKV-E protein as the coated antigen.

To develop a double-antibody sandwich ELISA (DAS-ELISA) for CHIKV detection, the effectiveness of different combinations of capture Nb-Fcs and HRP–Nb-Fcs was analyzed using a square matrix method. Serial dilution tests were used to determine the optimal working dilutions of the capture Nb-Fc and HRP–Nb-Fc. Briefly, the selected Nb-Fc was serially diluted from 4 µg/mL to 0.5 µg/mL in sodium bicarbonate buffer, coated onto 96-well ELISA microplates (100 μL/well) and incubated overnight at 4°C. After three rinses with PBST and blocking with 0.01 M PBS containing 3% BSA (200 μL/well). 100 ng/mL of CHIKV-E protein was added and incubated for 1 h, with PBS used as the negative control.

After washing with PBST, HRP–Nb-Fc was added at different dilution ratios (1:1,000 to 1:32,000) and incubated for 1 h. After washing with PBST, TMB substrate was added to each well and reacted for 10 min. The optical density at 450–630 nm (OD_450-630_) was immediately measured after termination with 2 M H_2_SO_4_. The combination yielding a negative control OD_450-630_≦0.05 and a positive well with the highest OD_450–630_ value was selected as the optimal antibody concentration.

The cutoff value was defined as the OD_450–630_ value×2.1 of the negative controls. Hence, samples giving OD_450–630_ values higher than 2.1×negative controls were recorded as positive.

### 2.7 Development of AuNP-ICTS for CHIKV detection

In this assay, gold nanoparticle (AuNP) solution was synthesized using trisodium citrate to reduce HAuCl_4_·3H_2_O. Firstly, 100 mL of 0.01% HAuCl_4_·3H_2_O was boiled, and 2 mL of 1% trisodium citrate was added and kept boiling for 20 min. The resulting clear wine-red AuNP solution was diluted to 100 mL with distilled water.

The diameter and size distribution of the synthesized AuNP were characterized by dynamic light scattering (DLS) using a Zetasizer Nano ZS90 system (Malvern Panalytical, UK). Measurements were conducted at 25°C in disposable sizing cuvettes. Data from three consecutive measurements were analyzed using Zetasizer software (version 8.02) to obtain the Z-average diameter and polydispersity index (PDI).

As the AuNP-based immunochromatographic test strip (AuNP-ICTS) is designed for detecting targets in human serum, the control line was coated with goat anti-mouse IgG antibodies. Consequently, the human IgG1-Fc domain of the recombinant nanobody used as the AuNP-labeled detector was replaced with mouse IgG1-Fc (mIgG1-Fc) to serve as a specific target, thereby ensuring a reliable control signal. For the synthesis of AuNP-labeled Nb–mIgG1-Fc, the pH of the AuNP solution was adjusted to 7.5 by adding 0.2 M K_2_CO_3_. Subsequently, 0.5 mg of purified Nb–mIgG1-Fc (1 mg/mL) was slowly added dropwise to 50 mL of AuNP solution, and the mixture was gently stirred for 50 min at room temperature (RT). Then, 2.5 mL of 10% BSA solution (w/v, g/mL) was slowly added dropwise to the AuNP/Nb–mIgG1-Fc solution, followed by gentle stirring for another 50 min at RT. The mixture was then centrifuged at 12,000 rpm for 20 min at 4°C. After discarding the supernatant containing unconjugated Nb–mIgG1-Fc, the resulting AuNP–Nb–mIgG1-Fc pellets were dissolved in 5 mL of 0.01 M PBS (pH 7.4) containing 3% sucrose and 2% BSA, then stored at 4°C until further use.

The AuNP-ICTS consists of a sample pad, conjugate pad, nitrocellulose membrane, and absorbent pad. The sample pad (DL42, Kinbio Biotechnology, Shanghai, China) made of glass fibers was pretreated with blocking buffer (0.01 M PBS containing 3% BSA, pH 7.4) and dried at 37°C for 3 h. Another Nb-Fc (1.5 mg/mL) and goat anti-mouse IgG antibodies (2 mg/mL) were sprayed onto the nitrocellulose membrane (Sartorius AG. Millipore, Billerica, MA, USA) and used as the test (T) line (1 µL/cm) and control (C) line (1 µL/cm), respectively. The prepared AuNP–Nb–mIgG1-Fc conjugates were sprayed onto the conjugate pad (RB65, Kinbio Biotechnology, Shanghai, China).

Subsequently, all the above components and the absorbent pad (CH27, Kinbio Biotechnology, Shanghai, China) were assembled sequentially onto a polyvinyl chloride (PVC) backing (SMNF-31, Kinbio Biotechnology, Shanghai, China) with a 2 mm overlap between each adjacent component. Finally, the assembled strips were cut into 3.5 mm-wide strips and stored in a desiccated container for further use.

### 2.8 Detection of CHIKV-E in PBS and human serum

To analyze the detection performance of the developed DAS-ELISA and AuNP-ICTS, CHIKV-E protein and several related viral proteins were tested. These included envelope proteins from four other alphaviruses—MAYV, EEEV, VEEV, and WEEV—as well as two flaviviruses, DENV (dengue virus) and ZIKV (Zika virus).

All envelope proteins were diluted to a working concentration of 1 µg/mL in 0.01 M PBS and in human negative serum, respectively. The expression and purification methods for MAYV-E, EEEV-E, VEEV-E, and WEEV-E proteins were identical to those used for CHIKV-E protein. DENV-E and ZIKV-E proteins were purchased from MedChemExpress (Monmouth Junction, NJ, USA).

Human serum was obtained from five healthy volunteers (aged 25–35 years) after informed consent.

### 2.9 Prediction of the conformation of Nbs with CHIK-E protein

An experimentally determined CHIKV antigen structure (genotype S37997; PDB 3J2W; 5.0 Å resolution) served as the receptor, and the ligand was the nanobody (N055 or 10G4) structure modeled using IgFold (42). Global protein–protein docking with HDOCK (43) generated 1,000 candidate complexes.

The top 20 candidate complexes were retained, redundant poses were removed by interface-based clustering, and conformations with severe steric clashes were discarded. For cross-validation, we used AlphaFold3 (44) to directly model the antigen–nanobody complex and record the inter-protein TM score (ipTM). Antigen residues within 4.5 Å of any antibody atom were designated as the epitope; this epitope was mapped onto the HDOCK candidates, retaining only poses with epitope overlap. The remaining poses were ranked by the HDOCK scoring function, and the highest-scoring pose was retained as the final complex structure.

## 3 Results

### 3.1 Preparation of CHIKV-E protein

The gene sequence of the CHIKV-E protein was artificially synthesized and subsequently expressed using the insect cell–baculovirus system. The recombinant CHIKV-E protein was purified via nickel affinity chromatography. SDS–PAGE analysis was performed to assess the expression level and purity of the purified CHIKV-E protein.

**Figure 2:**
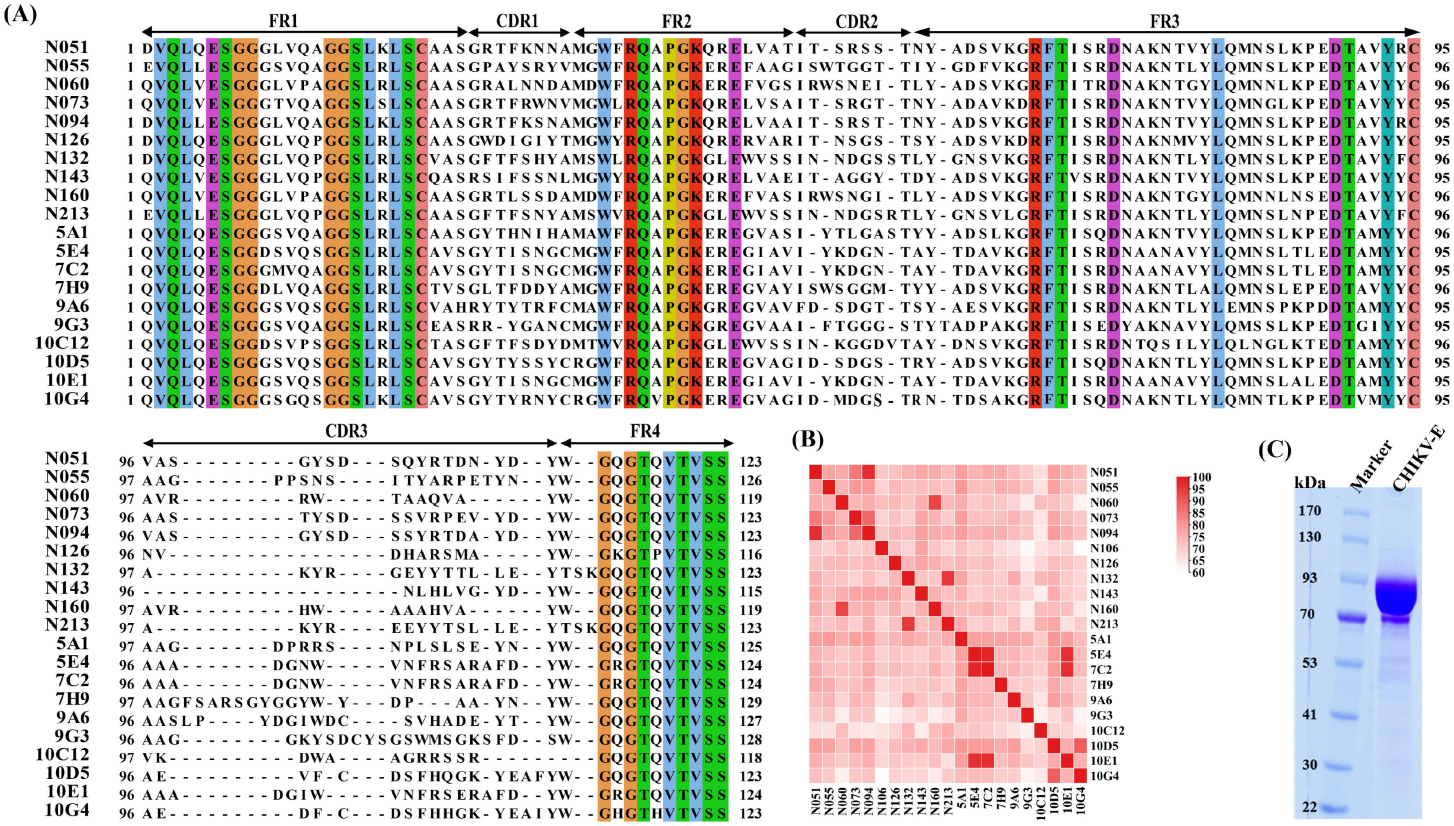
Screening of CHIKV-E–specific nanobodies from an immune nanobody library. **(A)** Alignment of the amino acid sequences and CDR region prediction of 20 anti-CHIKV Nbs. **(B)** Similarity analysis of the amino acid sequences of 20 anti-CHIKV Nbs. **(C)** SDS–PAGE of purified CHIKV-E protein.

The results showed that the purified CHIKV-E protein exhibited a molecular weight of approximately 100 kDa (**Figure 2C**), which is consistent with the expected size. Furthermore, the purity of the protein met the required standards for its use as an immunogen.

### 3.2 Preparation of anti-CHIKV Nb-Fcs

The immune Nb library was successfully constructed by immunizing an alpaca with purified CHIKV-E protein as an immunogen. Twenty highly sensitive anti-CHIKV Nbs were retrieved from the immune Nb library through two rounds of screening by phage display under stringent conditions. The core coding sequences of these 20 Nbs were determined by sequencing.

The complementarity-determining region (CDR) sequence analysis and similarity analysis of all Nb sequences are shown in **Figures 2A, B**. The data revealed significant diversity in their CDRs, particularly in the CDR3 regions, which showed remarkable length variation (9–22 residues) and sequence heterogeneity.

**Figure 3:**
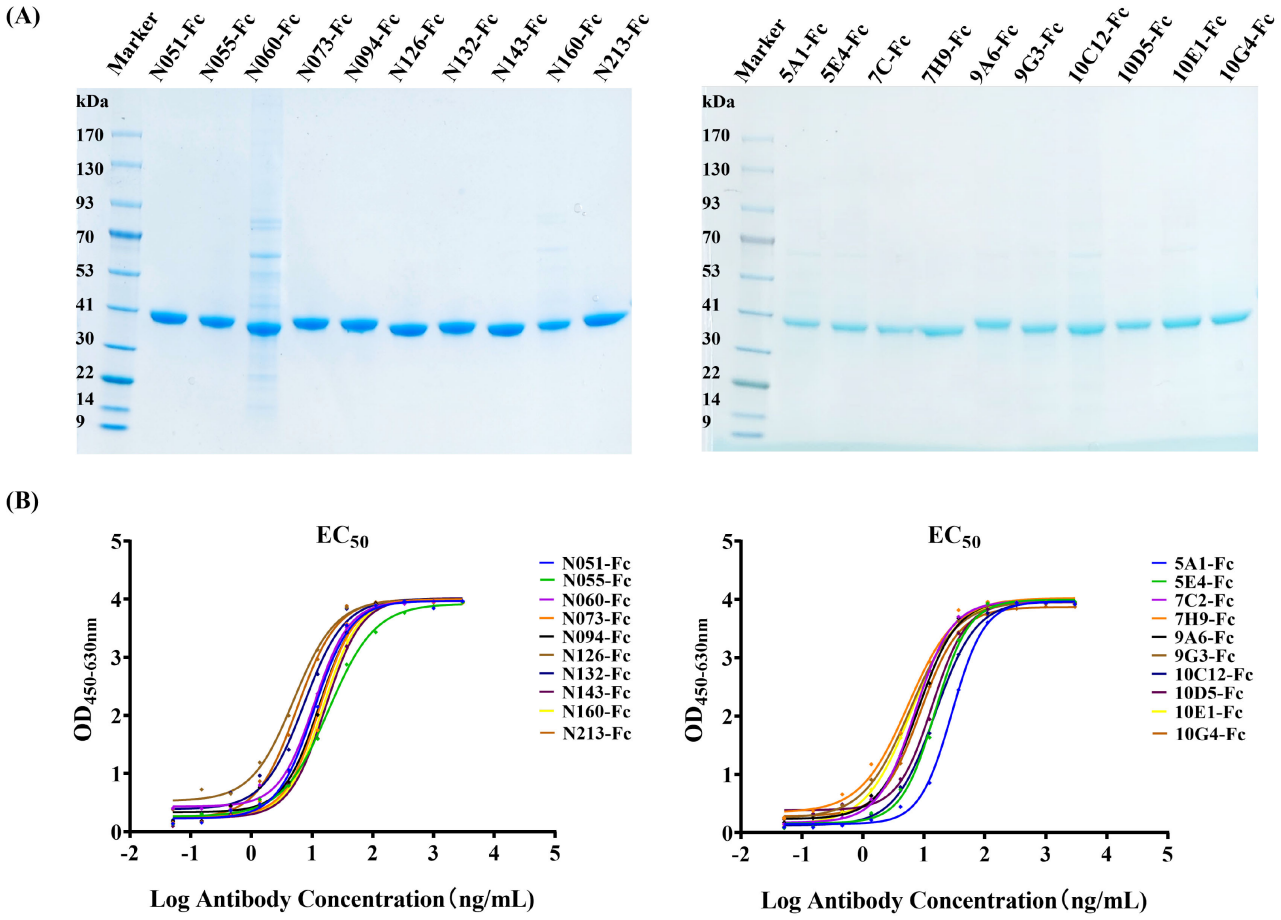
Characterization of anti-CHIKV Nb-Fcs. **(A)** SDS–PAGE of purified Nb-Fcs. Twenty anti-CHIKV Nb-Fcs were purified using Protein A columns. **(B)** Binding activity of Nb-Fcs to immobilized CHIKV-E protein quantified by indirect ELISA.

The human IgG1-Fc sequence was added to the C-terminal of each Nb sequence, and the recombinant sequence was cloned into the pcDNA3.4 vector. The recombinant antibody (Nb-Fc) was successfully expressed using the Expi293F cell expression system. Expression and purity of the Nb-Fcs were analyzed by SDS–PAGE under reducing conditions (**Figure 3A**). All purified Nb-Fcs migrated as single bands at approximately 40 kDa, corresponding to their predicted molecular weights.

### 3.3 Characterization of anti-CHIKV Nb-Fcs

Indirect ELISA results showed that all Nb-Fcs could bind to the CHIKV-E antigen in a dose-dependent manner (**Figure 3B**). When the concentration of coated CHIKV-E was 2 µg/mL, the maximum signal response (plateau phase) of the ELISA reaction was reached when each Nb-Fc was diluted to approximately 100 ng/mL, indicating that the Nb-Fcs exhibited strong binding activity to CHIKV-E protein.

The EC_50_ (half-maximal effective concentration) values of the 20 Nb-Fcs to CHIKV-E protein were evaluated and calculated using the four-parameter logistic model and are presented in **Table 1**. The EC_50_ values (5.00–28.79 ng/mL) indicate modest variations in binding capacity among these Nb-Fcs, likely attributable to structural diversity in their CDR3 regions (length range: 9–22 residues).

**Table 1:** EC_50_ (half-maximal effect concentration) of purified Nb-Fcs.

Nb-Fc	EC_50_ (ng/mL)	Nb-Fc	EC_50_ (ng/mL)
N051-Fc	10.74	5A1-Fc	28.79
N055-Fc	18.65	5E4-Fc	15.30
N060-Fc	10.81	7C2-Fc	7.15
N073-Fc	13.37	7H9-Fc	5.48
N094-Fc	13.13	9A6-Fc	8.02
N126-Fc	5.00	9G3-Fc	5.90
N132-Fc	7.37	10C12-Fc	15.39
N143-Fc	16.43	10D5-Fc	13.60
N160-Fc	15.03	10E1-Fc	6.13
N213-Fc	5.62	10G4-Fc	8.78

### 3.4 Development of DAS-ELISA for CHIKV detection

**Figure 4:**
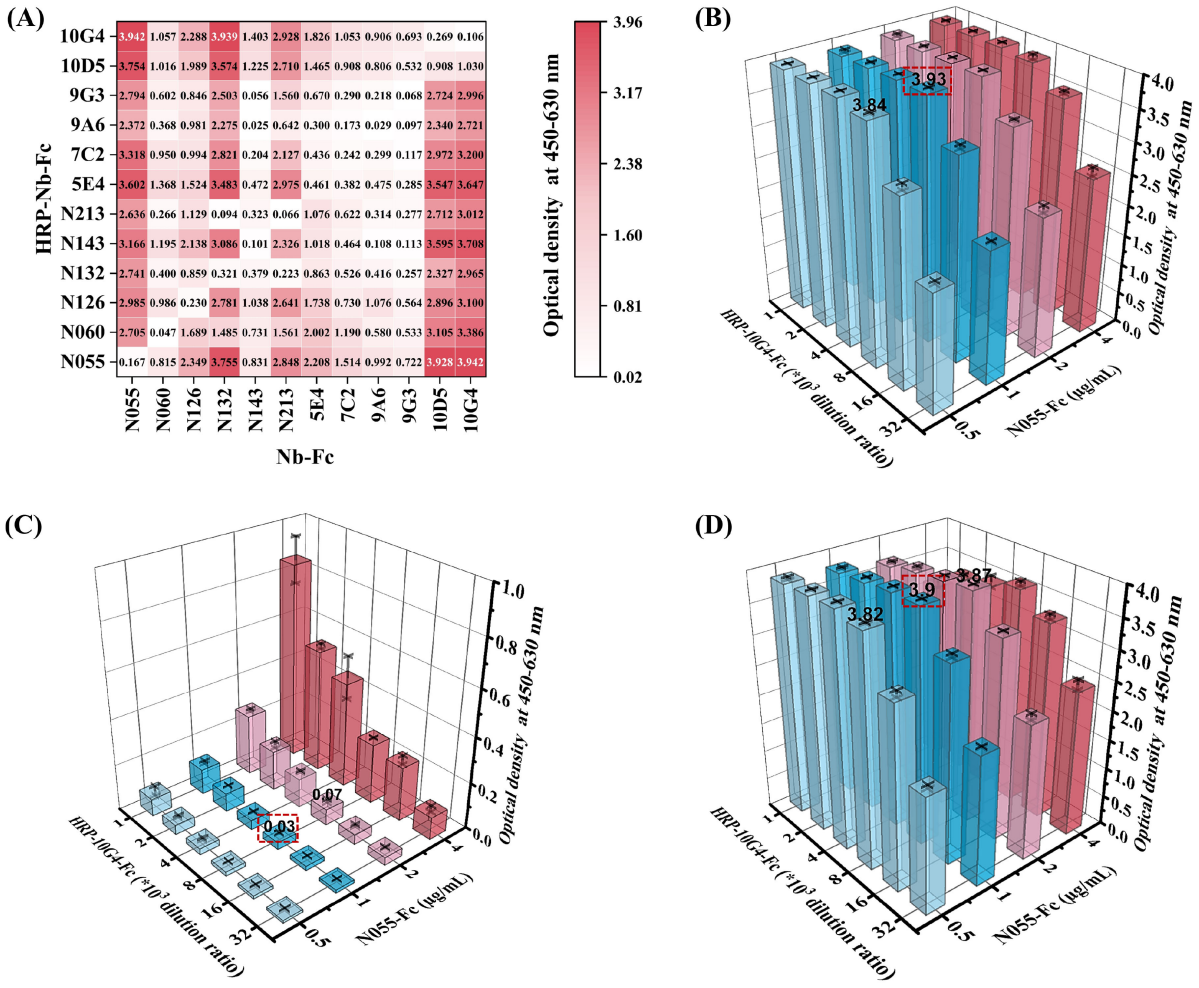
Establishment and optimization of DAS-ELISA for CHIKV detection. **(A)** Heatmap of pairwise screening of twelve Nb-Fcs and their corresponding HRP–Nb-Fcs. Twelve Nb-Fcs were coated onto 96-well plates at 1 µg/mL. CHIKV-E protein (100 ng/mL) and 0.01 M PBS were used as positive and negative samples, respectively. Twelve corresponding 8000-fold dilutions of HRP–Nb-Fcs were used as labeled antibodies for determining OD_450_–_630_. Values represent the average OD_450_–_630_ of positive wells minus negative wells for different antibody combinations. **(B–D)** Optimal working dilutions of N055-Fc and HRP-10G4-Fc for DAS-ELISA. **(B)** OD_450_–_630_ values of positive samples with different N055-Fc and HRP-10G4-Fc concentration combinations. **(C)** OD_450_–_630_ values of negative samples with different N055-Fc and HRP-10G4-Fc concentration combinations. **(D)** OD_450_–_630_ values of positive minus negative samples. Bars represent corresponding OD_450_–_630_. The red-boxed combination represents the optimal working concentrations of N055-Fc and HRP-10G4-Fc. Data represent the mean ± SD from two biological replicates; the experiment was repeated three times.

Nb-Fcs anti-CHIKV were conjugated with horseradish peroxidase (HRP) using an HRP Labeling Kit, and twelve HRP-Nb-Fcs with a titer of at least 1×10^7^ were screened by indirect ELISA (**Table 2**). The result of the square-matrix assays showed that DAS-ELISA using N055-Fc and 10G4-Fc antibodies gave the best detection effectiveness for CHIKV-E. The N055-Fc/10G4-Fc combination yielded the highest OD_450–630_ values for detecting the same concentration of CHIKV-E protein (**Figure 4A**). Notably, while neither N055-Fc (EC_50_: 18.65 ng/mL) nor 10G4-Fc (EC_50_: 8.78 ng/mL) exhibited the highest individual binding affinity (**Table 1**), their combined use achieved superior detection sensitivity. These results suggest that DAS-ELISA performance depends not only on monomeric affinity but also on epitope accessibility and binding non-competition.

**Table 2:** The titer of HRP-Nb-Fc to CHIKV-E.

HRP-Nb-Fc	titer	HRP-Nb-Fc	titer
N055-Fc	1×10^7^	5E4-Fc	1×10^7^
N060-Fc	1×10^7^	7C2-Fc	1×10^7^
N126-Fc	1×10^7^	9A6-Fc	1×10^7^
N132-Fc	1×10^7^	9G3-Fc	1×10^7^
N143-Fc	1×10^7^	10D5-Fc	2×10^7^
N213-Fc	2×10^7^	10G4-Fc	2×10^7^

Considering that the titer of labeling antibody HRP-10G4-Fc was higher than that of HRP-N055-Fc, N055-Fc was used as the capture antibody and HRP-10G4-Fc as the detection antibody. The serial-dilution test of DAS-ELISA showed that the optimal coating concentration of N055-Fc was 1 µg/mL, and the optimal working dilution of the detection antibody HRP-10G4-Fc was 1:8000 (**Figures 4B–D**).

### 3.5 Specificity and sensitivity of the DAS-ELISA for CHIKV detection

**Figure 5:**
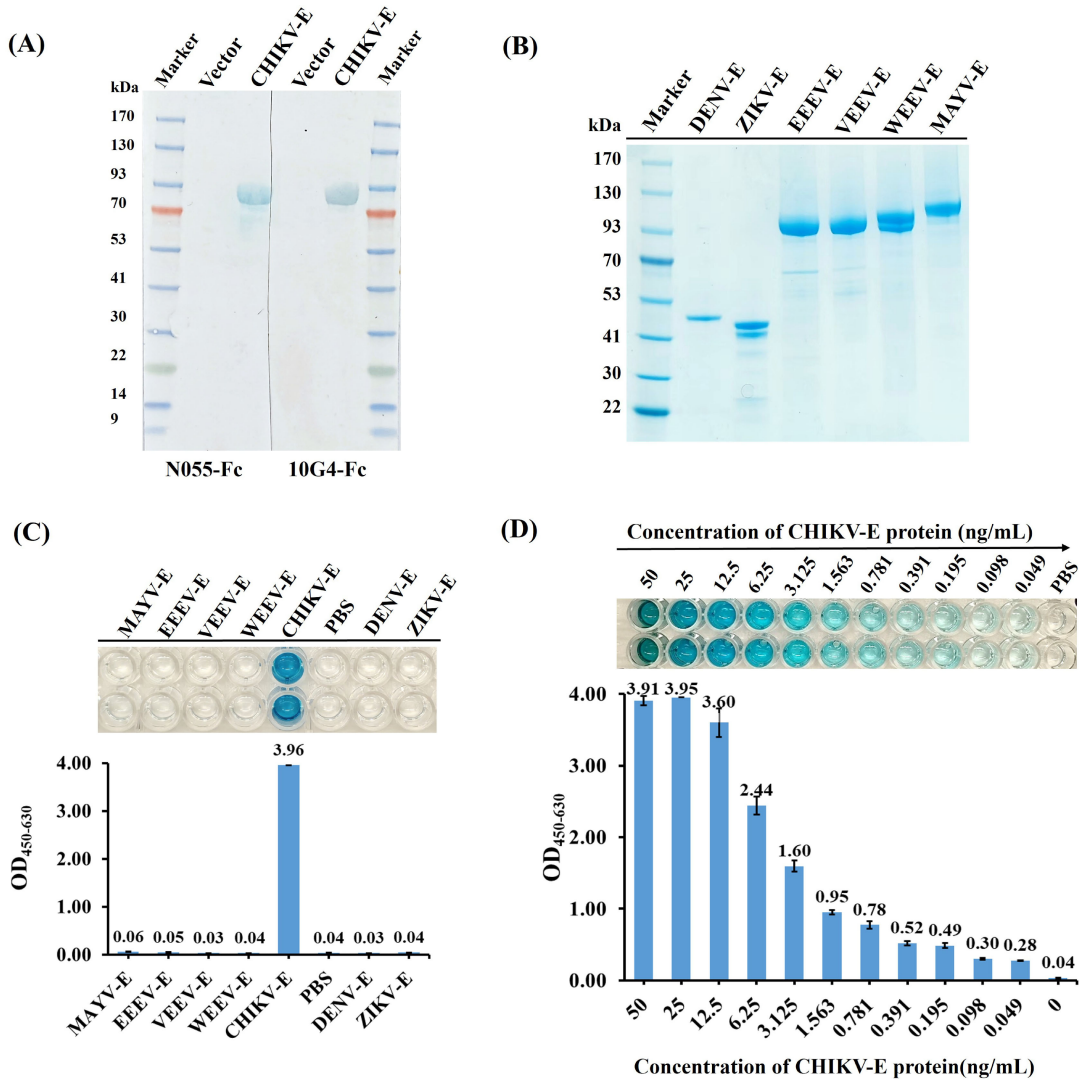
Specificity and sensitivity analyses of the DAS-ELISA for CHIKV detection. **(A)** Epitope verification of antigen binding to N055-Fc and 10G4-Fc by western blot using CHIKV-E protein and empty vector protein as samples. **(B)** SDS–PAGE of envelope proteins of CHIKV, EEEV, VEEV, WEEV, MAYV, DENV, and ZIKV. Expression and purification methods for EEEV-E, VEEV-E, WEEV-E, and MAYV-E were consistent with CHIKV-E. ZIKV-E (aa F257–G694, Accession ALU33341, His-tag) and DENV-E (aa F247–G675, Accession AAC59274, His-tag) were purchased from MedChemExpress. **(C)** Specificity analyses of DAS-ELISA using CHIKV-E, EEEV-E, VEEV-E, WEEV-E, MAYV-E, DENV-E, and ZIKV-E proteins as test samples. PBS served as the negative control. **(D)** Sensitivity analyses of DAS-ELISA for CHIKV detection. CHIKV-E protein was serially diluted from 50 ng/mL to 49 pg/mL in 0.01 M PBS; 100 µL of each dilution was tested. PBS served as the negative control. Each sample was tested in duplicate; data are presented as mean ± SD. Three independent experiments were performed.

Western blot was performed to determine the binding epitopes of N055-Fc and 10G4-Fc to CHIKV-E protein, and the results demonstrated that the two Nb-Fcs reacted specifically and strongly with CHIKV-E protein, but not with the empty His-tag vector protein (**Figure 5A**); that is, the antigenic epitopes recognized by the two antibodies are linear rather than conformational.

The specificity of the DAS-ELISA for CHIKV was evaluated by testing CHIKV-E protein alongside envelope proteins from related alphaviruses (MAYV, EEEV, VEEV, WEEV) and flaviviruses (DENV, ZIKV). As expected, the DAS-ELISA showed that wells containing CHIKV-E protein had strong positive reactions, but not those with MAYV-E, EEEV-E, WEEV-E, VEEV-E, DENV-E, or ZIKV-E proteins, nor the PBS negative control (**Figures 5B, C**). This suggests that the developed DAS-ELISA can specifically detect CHIKV.

The sensitivity of the DAS-ELISA was further analyzed using CHIKV-E protein serially diluted from 50 ng/mL to 49 pg/mL in PBS. The assay successfully detected CHIKV-E protein even at the lowest concentration of 49 pg/mL (**Figure 5D**), which was thus established as the limit of detection (LoD). These results demonstrate that the developed DAS-ELISA exhibits high sensitivity and specificity for CHIKV detection.

### 3.6 Epitope mapping of N055 and 10G4 nanobodies against CHIKV-E protein

AlphaFold3 modeling of the antigen–nanobody complexes yielded ipTM scores of 0.68 and 0.69, indicating a reliable predicted protein–protein interaction interface. Accordingly, we defined the antigenic contact surface within this interface as the epitope and, among HDOCK candidates consistent with this epitope, selected the final models according to their HDOCK scores.

**Figure 6:**
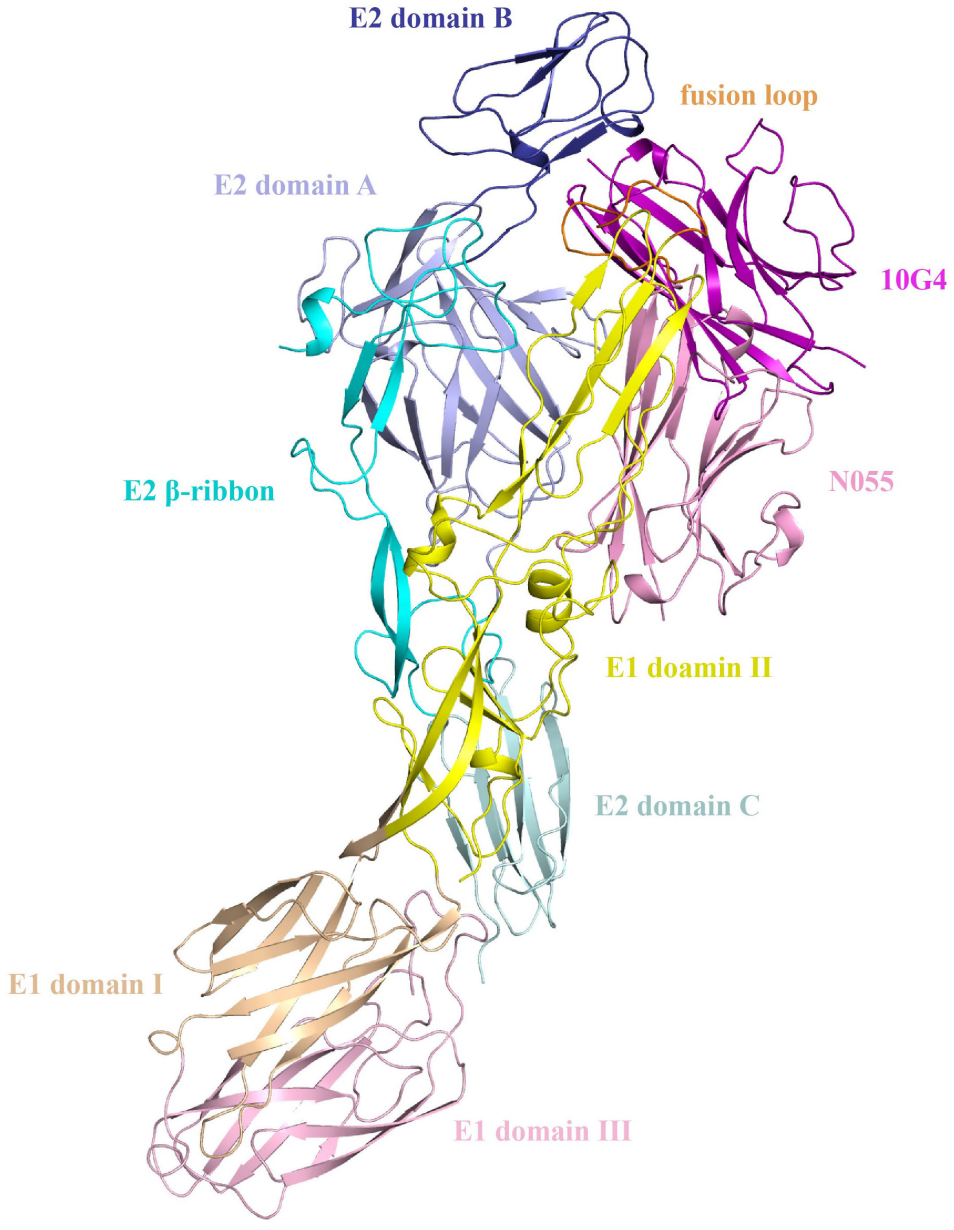
Structural docking models of nanobodies N055 and 10G4 bound to CHIKV-E protein.

For nanobody N055, candidate 19 in the original HDOCK ranking was selected (HDOCK score: −301.85). Epitope-mapping analysis revealed that the epitope primarily localizes to E1 Domain II. For nanobody 10G4, candidate 3 (HDOCK score: −334.52) was selected, demonstrating a complex binding pattern that targets the E1 fusion loop while simultaneously binding both E1 Domain II and E2 Domain B (**Figure 6**). The epitope-mapping data indicated that the two nanobodies, recognizing distinct antigenic determinants, were suitable for establishing a double-antibody sandwich ELISA (DAS-ELISA) assay.

### 3.7 Detection of CHIKV in human serum samples

**Figure 7:**
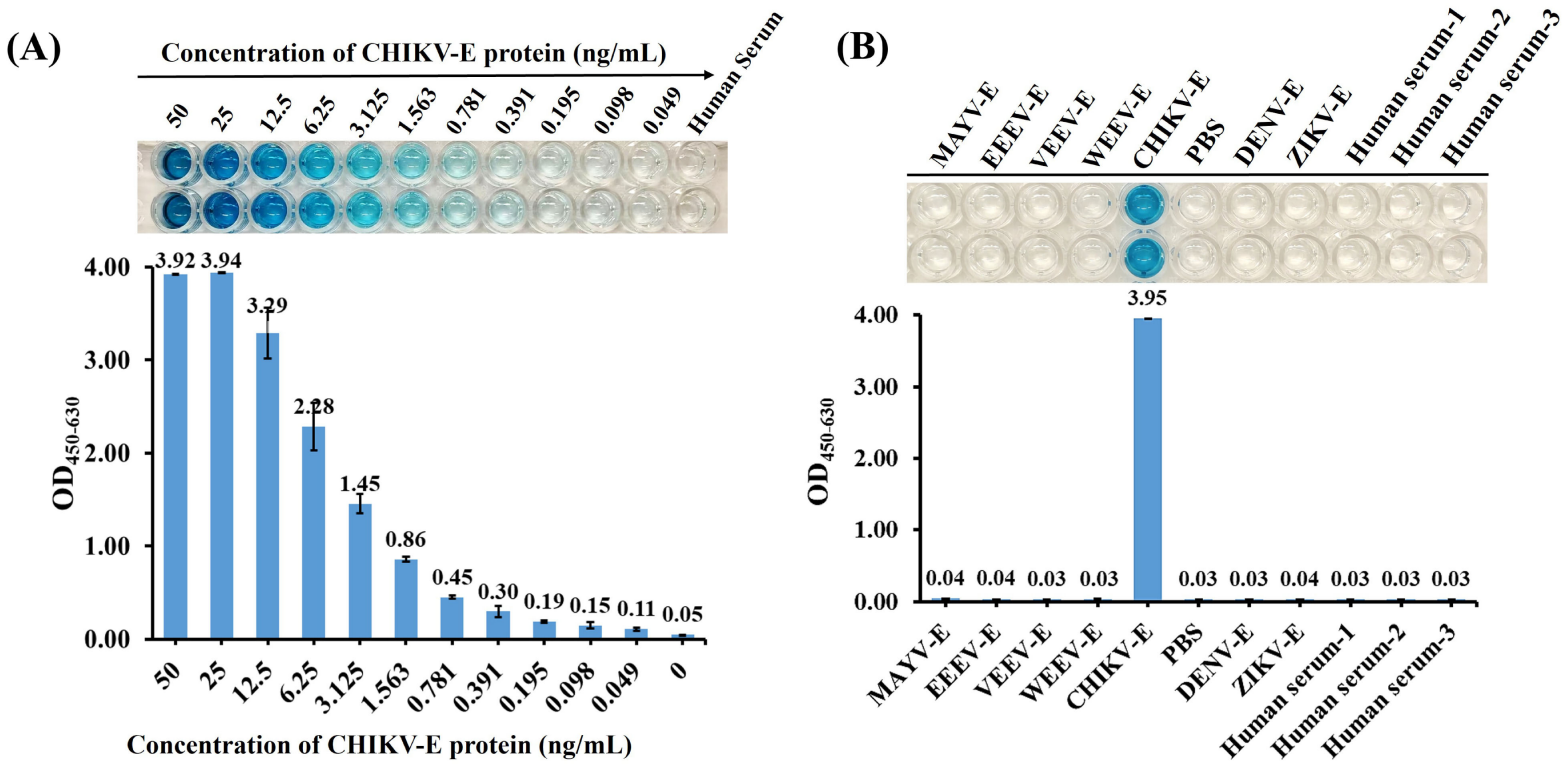
Detection of CHIKV-E in human serum samples. **(A)** Sensitivity analyses of the DAS-ELISA. CHIKV-E protein was serially diluted from 50 ng/mL to 49 pg/mL using negative human serum; 100 µL of each dilution was tested. Negative human serum served as the control. **(B)** Specificity analyses of the DAS-ELISA using CHIKV-E, EEEV-E, VEEV-E, WEEV-E, MAYV-E, DENV-E, and ZIKV-E proteins in negative human serum, along with multiple negative human-serum samples. PBS was used as the negative control. Data represent the mean ± SD of two biological replicates; three independent trials were conducted.

The CHIKV-E protein was diluted from 50 ng/mL to 49 pg/mL in a two-fold gradient using negative human serum, with negative human serum as the control, to analyze the sensitivity of the DAS-ELISA in detecting CHIKV antigen in real human serum. The results showed that the sensitivity of the DAS-ELISA for detecting CHIKV envelope protein in real human serum could still reach 49 pg/mL (**Figure 7A**).

In addition, the established DAS-ELISA showed no cross-reactivity in human serum with a diverse panel of samples, including envelope proteins from other alphaviruses (MAYV, EEEV, VEEV, and WEEV), co-circulating flaviviruses (DENV and ZIKV), and negative human-serum controls (**Figure 7B**), validating its effectiveness for specific detection of CHIKV in human serum.

### 3.8 Performance of the AuNP-ICTS for CHIKV detection

**Figure 8:**
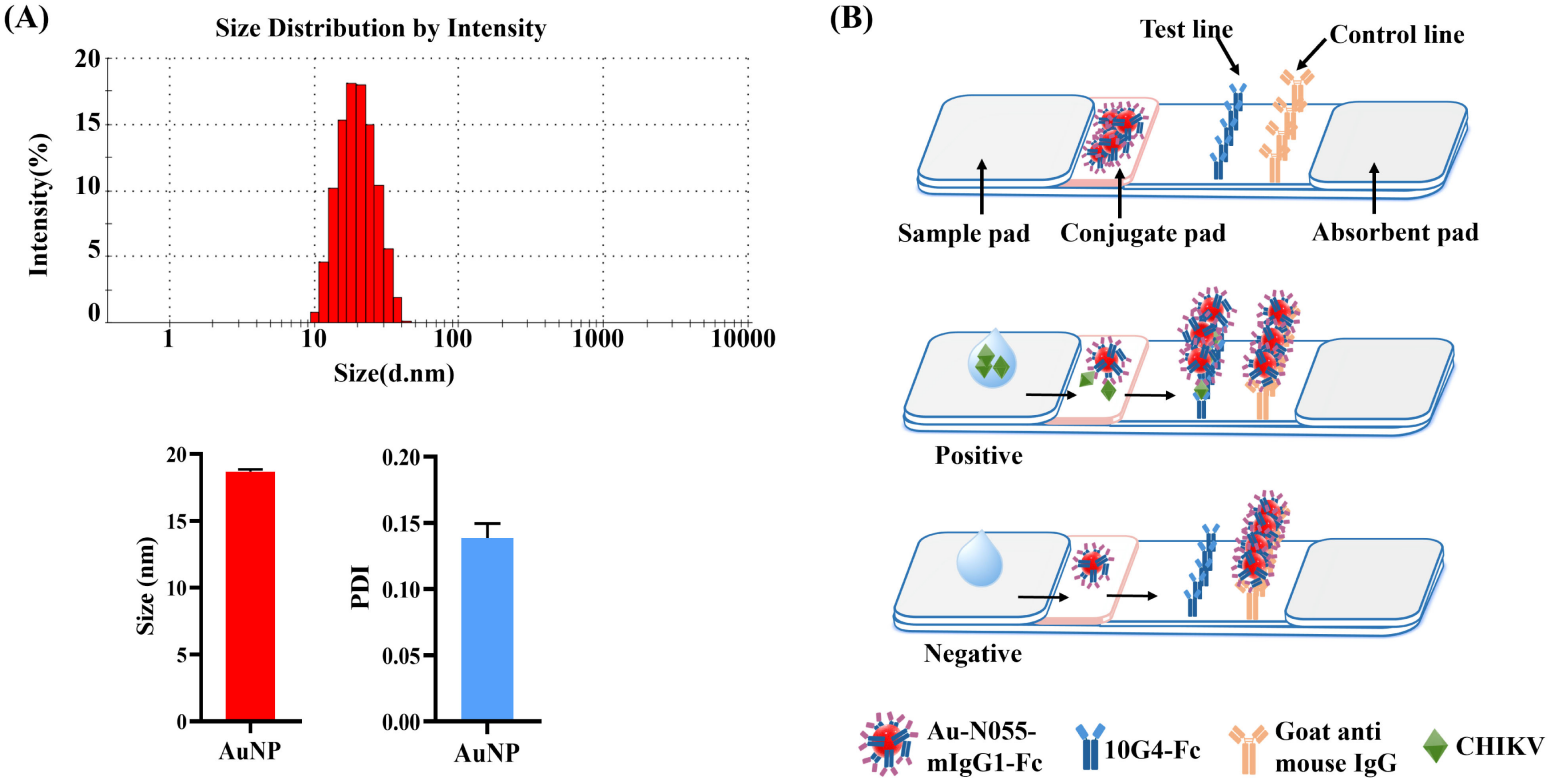
Fabrication and principle of the AuNP-ICTS for CHIKV detection. **(A)** Characterization of synthesized AuNPs by dynamic light scattering (DLS). **(B)** Schematic diagram of AuNP-ICTS for CHIKV detection.

The synthesized AuNPs exhibited an average diameter of 20 nm as characterized by DLS. The DLS measurement revealed a low polydispersity index (PDI < 0.2), indicating a narrow size distribution (**Figure 8A**). Based on the selected antibodies used in the developed DAS-ELISA, the AuNP-ICTS was established using antibodies N055-mIgG1-Fc and 10G4-Fc. The AuNP-ICTS was developed as a double-antibody sandwich-format immunoassay.

When the analyte CHIKV was present, it bound with AuNP-N055-mIgG1-Fc to form the immunocomplex AuNP-N055-mIgG1-Fc–CHIKV. The immunocomplex migrated forward, and CHIKV was captured by immobilized 10G4-Fc on the T line to form a red line, while free AuNP-N055-mIgG1-Fc was captured by goat anti-mouse IgG on the C line to form another red line. For negative samples, AuNP-N055-mIgG1-Fc flowed across the T line with no colorimetric signal and was captured only on the C line (**Figure 8B**).

**Figure 9:**
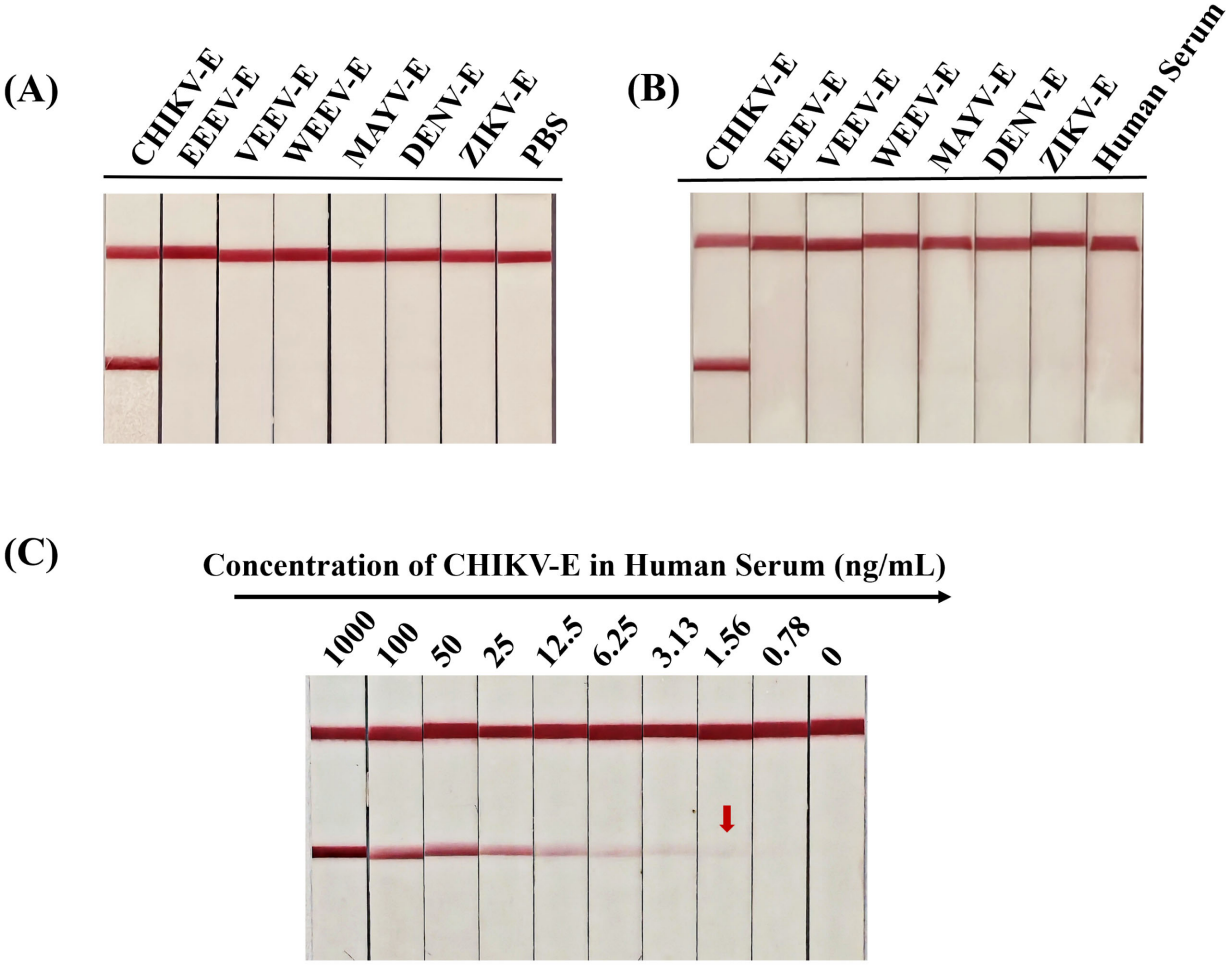
Specificity and sensitivity analyses of the developed AuNP-ICTS for CHIKV detection. **(A)** Specificity of the developed test strip determined using CHIKV-E, EEEV-E, VEEV-E, WEEV-E, MAYV-E, DENV-E, and ZIKV-E proteins in PBS; PBS served as the negative control. **(B)** Specificity of the developed test strip determined using CHIKV-E, EEEV-E, VEEV-E, WEEV-E, MAYV-E, DENV-E, and ZIKV-E proteins in negative human serum; human serum served as the negative control. **(C)** Sensitivity of the developed test strips determined using CHIKV-E protein diluted with human serum from 1000 ng/mL to 0.78 ng/mL. Human serum served as the negative control. Each experiment was repeated three times.

The specificity of AuNP-ICTS was determined using envelope proteins of alphaviruses (CHIKV, EEEV, VEEV, WEEV, MAYV) and two co-circulating flaviviruses (DENV and ZIKV) in PBS and negative human serum. When samples containing CHIKV-E protein were added to the sample pads, two red lines appeared on both the C and T lines within 10 min (**Figures 9A, B**). For other samples, only red deposition appeared on the C line, with no colorimetric signal on the T line.

Sensitivity analysis of the AuNP-ICTS revealed a limit of detection of 1.56 ng/mL for CHIKV-E protein in human serum, as a visible test line was still observed at this concentration (**Figure 9C**). Although the detection limit of the AuNP-ICTS (1.56 ng/mL) was approximately 32 times higher than that of the DAS-ELISA (49 pg/mL), the AuNP-ICTS provided rapid results within 10 min while maintaining equipment-free operation. Moreover, the strip’s LoD was still higher than that of other existing point-of-care assays. These results indicated that the developed AuNP-ICTS has excellent specificity and sensitivity for CHIKV monitoring.

## 4 Discussion

CHIKV is rapidly spreading through many countries as global climate change and globalization have led to the expansion of mosquito vectors into new regions (26, 27). Moreover, there is currently no approved specific antiviral treatment available for CHIKV, and treatment of infected patients mainly relies on the use of widely employed anti-inflammatory drugs and disease-modifying antirheumatic drugs (DMARDs) for symptomatic relief. In addition, most preclinical vaccines are still under development, and only a few vaccine candidates are under clinical trial evaluation worldwide. The lack of preventive vaccines and approved antiviral treatments is turning CHIKV into a major global health threat. At present, the prevention and control of CHIKV mainly rely on virus quarantine, surveillance, and vector control. Therefore, early antigen detection and diagnosis are essential and will facilitate surveillance and more effective clinical management of CHIKV infections.

The detection techniques for CHIKV mainly include RT-PCR–based molecular methods and serological methods based on CHIKV-specific IgM and IgG antibodies (28, 30, 45, 46). Commercially available nucleic acid tests for CHIKV are not widely used because of cost, equipment requirements, and technical expertise. Compared with PCR-based molecular assays, serological techniques are generally considered the most practical and cost-effective methods for virus monitoring due to their operational simplicity. Serum IgM and IgG are still the most commonly used biomarkers for CHIKV detection. Serum IgM appears at a relatively early stage of infection—about 1 week after onset—and serves as a diagnostic index for acute infection, while serum IgG can be detected at least 1 week after the onset of acute infection (13). According to the high correlation between serological assays based on antigen detection and PCR-based molecular methods, antigen-based serological detection could achieve early virus detection by specifically recognizing viral proteins and overcoming the window-period limitation of IgM/IgG detection.

To date, several antigen-based serological assays have been reported for CHIKV detection (**Table 3**). Kim et al. prepared anti-CHIKV-E2 monoclonal antibody 19-1, which can be used for the detection of CHIKV. The 19–1 mAb showed higher binding affinity to various amounts of CHIKV-E2 protein (1.2–312.5 ng/mL in PBS or 19.5–5000 ng/mL in 1% human serum) compared with other commercially available mAbs (31). Li et al. established a sandwich ELISA using multivalent VHH antibodies Nb-2E8 and Nb-3C5, which showed high affinity with CHIKV-E2 protein in the 5–1000 ng/mL range (34). In another study, a fluorescence-linked immunosorbent assay (FLISA) based on two mAbs (2B5 and 2C8) targeting CHIKV-E1 protein was developed. Based on optical density values, the limits of detection of the 2B5 and 2C8 antibody pair-linked ELISA and FLISA were both 4.88 ng/mL for CHIKV-E1 in distilled water (33). Recently, a sandwich TRFIA detection method using peptide aptamer–antibody pairs was applied to detect CHIKV; the detection limits of peptide aptamers B2 and B3 for CHIKV-E2 protein were 8.5 ng/mL and 164 ng/mL, respectively, in PBS. Peptide aptamer B2 was more suitable for clinical specimen testing, with a detection limit of 57.8 ng/mL in 1:10 diluted serum samples (35). While ELISA-based methods remain widely used, their reliance on multiple washing steps and relatively long incubation times limits their utility in rapid diagnostics. A rapid and simple test tool for CHIKV detection is crucial for effective viral management measures. Reddy et al. developed a rapid lateral flow E1/E2 antigen test and ELISA, with detection limits ranging from 37.08 to 844.16 ng/mL—within the viral load concentrations found in acute CHIKV patients (32). Another similar study demonstrated that a rapid immunochromatographic assay achieved a detection limit of 1×10^5^ PFU/mL for CHIKV, with diagnostic performance satisfying clinical requirements for acute-phase chikungunya fever detection. These advancements highlight the importance of rapid, simple diagnostic tools for effective CHIKV management.

**Table 3:** Antigen-based methods for CHIKV detection.

Method	Target of the assay	Limit of detection	Sample matrix	Reference
ELISA	CHIKV-E2	E2: 1.2 ng/mL E2: 19.5 ng/mL	PBS; 1% human serum	(31)
ELISA	CHIKV-E1	E1: 4.88 ng/mL; CHIKV (virion): 2 × 10^5^ PFU/mL	distilled water	(33)
FLISA	CHIKV-E1	E1: 4.88 ng/mL; CHIKV (virion): 1 × 10^5^ PFU/mL	distilled water	(33)
ELISA	CHIKV-E2	E2: 5–1000 ng/mL	PBS	(34)
TRFIA	CHIKV-E2	E2: 8.5 ng/mL; E2: 57.8 ng/mL	PBS 1:10 diluted serum	(35)
ELISA	CHIKV-E1&E2	E1:133.48 ng/mL; E2:363.98 ng/mL	serum sample diluted in PBS containing 2.5% skimmed milk powder	(32)
Lateral Flow Assays/Dipstick	CHIKV-E1&E2	E1: 844.16/15.55 ng/mL; E2: 875.86/37.08 ng/mL		(32)
Immuno-chromatographic Test	CHIKV-E1	CHIKV (virion):1×10^5^ PFU/mL	healthy donor serum	(36)

In this work, 20 high-affinity anti-CHIKV Nbs were prepared using an immune Nb library with CHIKV-E protein as the immunogen. To enhance stability, recombinant Nbs (Nb-Fcs) were constructed by linking the human IgG1-Fc sequence to the C-terminus of the Nb core coding sequence. A highly sensitive DAS-ELISA for CHIKV detection was then developed based on two prepared Nb-Fcs (N055-Fc and 10G4-Fc). Additionally, another sensitive and visual detection method, AuNP-ICTS, was established using N055-mIgG1-Fc (mouse-derived IgG1-Fc) and 10G4-Fc. The specificities of the developed DAS-ELISA and AuNP-ICTS were verified by a positive reaction to CHIKV envelope protein but negative reactions to other alphaviruses (MAYV, EEEV, VEEV, WEEV) and two co-circulating flaviviruses (DENV and ZIKV). The detection sensitivities of the DAS-ELISA and AuNP-ICTS for CHIKV-E protein in human serum were 49 pg/mL and 1.56 ng/mL, respectively—significantly higher than other similar antigen detection methods. Moreover, the newly developed AuNP-ICTS could detect CHIKV within 10 min, showing significant potential for on-site CHIKV detection.

There are several potential limitations to this study. First, due to experimental constraints, clinical samples were not included. However, based on comparative analyses with existing methods, our assays demonstrate superior sensitivity for CHIKV-E protein detection relative to previously reported approaches. Although clinical validation remains to be conducted, these results suggest that our methods hold strong potential to improve both sensitivity and accuracy in clinical specimens. In subsequent studies, we will collect sufficient samples to validate the sensitivity and accuracy of the established DAS-ELISA and AuNP-ICTS methods.

Second, while detection sensitivity was significantly improved, the potential for hook effects in high-titer samples—a known limitation of DAS-ELISA—requires investigation. The clinical relevance of this phenomenon remains uncertain, as CHIKV viremia levels in human serum may not reach sufficient concentrations to induce hook effects. This aspect should be systematically evaluated in future clinical validations.

Third, although recombinant nanobody engineering has enhanced Nb stability, the long-term (6–12 month) stability of AuNP-ICTS under tropical conditions (high temperature and humidity) requires further assessment. Concurrently, the DAS-ELISA antibody reagent formulation needs optimization to improve stability for field deployment in CHIKV-endemic regions. Notably, our preliminary data demonstrate that both methods effectively detect envelope proteins across major CHIKV lineages (West African clade, WA; Asian clade; East/South/Central African clade, ECSA; and Indian Ocean lineage, IOL), confirming their broad-spectrum applicability (data not shown). This strongly suggests that the targeted epitopes are conserved among diverse CHIKV lineages, representing a significant advantage for diagnostic applications. We acknowledge that epitope variability could pose limitations if divergent strains emerge; thus, we will expand validation to include geographically diverse, recently circulating strains in future studies.

In conclusion, we screened 20 nanobodies from an immune phage library and developed two highly sensitive and specific assays (DAS-ELISA and AuNP-ICTS) for CHIKV antigen detection using the selected antibodies N055-Fc and 10G4-Fc. Our CHIKV-specific DAS-ELISA and AuNP-ICTS could serve as valuable tools for diagnosis, epidemiological surveillance, and quarantine inspection, aiding in the management of potential epidemics. Additionally, this work provides a reference for developing similar monitoring tools for other alphaviruses.

## Data Availability

The original contributions presented in the study are included in the article/Supplementary Material. Further inquiries can be directed to the corresponding authors.
